# Cross-Cultural Nomological Network of Gratitude: Findings From Midlife in the United States (MIDUS) and Japan (MIDJA)

**DOI:** 10.3389/fpsyg.2020.00571

**Published:** 2020-05-26

**Authors:** Tara Srirangarajan, Atsushi Oshio, Ayano Yamaguchi, Satoshi Akutsu

**Affiliations:** ^1^Department of Psychology, Stanford University, Stanford, CA, United States; ^2^Faculty of Letters, Arts and Sciences, Waseda University, Tokyo, Japan; ^3^College of Community and Human Services, Rikkyo University, Tokyo, Japan; ^4^School of International Corporate Strategy, Hitotsubashi University Business School, Tokyo, Japan

**Keywords:** gratitude, cross-cultural, nomological network, MIDUS, well-being, MIDJA

## Abstract

Gratitude enhances prosocial behavior and is considered a positive trait in most cultures, yet relatively little is known about its relationship to other psychological constructs, nor how it varies across diverse cultural contexts. To investigate the cross-cultural consistency of the benefits of having a grateful disposition, the current study examined the nomological network of gratitude in the United States and Japan, using data from two longitudinal studies: Midlife in the United States (MIDUS Refresher Biomarker Project) and Midlife in Japan (MIDJA). Results showed significant positive bivariate associations between trait gratitude and positive psychological functioning (Satisfaction with Life, Sympathy, Anger Control, Cognition Control, and Support/Affectual Solidarity Given to Relational Network) in both the United States and Japan. On the other hand, trait gratitude was negatively correlated with constructs associated with maladaptive psychological processes (Perceived Stress, Social Anxiety, Loneliness, and Anger-In) in both countries. The present findings provide valuable guidance for the development and implementation of future interventions that may lead to positive outcomes in individuals from diverse cultural and ethnic backgrounds.

## Introduction

Although gratitude has been extolled as a fundamental virtue in most societies, it has until recently been overshadowed by other constructs within the psychological literature. As the concept of gratitude has garnered increasing interest alongside the advent of positive psychology, scholars have made strides in demonstrating that gratitude is associated with enhanced well-being and may in fact serve as a buffer against maladaptive psychological functioning (Brown and Ryan, 2003; Brown et al., 2007; Keng et al., 2011). However, an open question remains: how might gratitude vary across cultures? Examining the similarities and differences in cultural values underlying the experience of gratitude may help to cultivate greater sensitivity toward the diversity of emotional experience and expression.

The importance of gratitude as a positive trait worth cultivating has been accepted throughout history and around the world. Although it may be a favorable characteristic that transcends cultural boundaries and holds long-lasting psychological benefits, this assumption has not been systematically tested using participants from diverse cultural backgrounds. In fact, previous research on gratitude has focused almost solely on the differences in the development and expression of gratitude within a Western context (Grant and Gino, 2010; Lambert and Fincham, 2011; Williams and Bartlett, 2015).

Of the few studies that do examine the concept of gratitude across different cultures, most solely examine the expression of gratitude (Pishghadam and Zarei, 2011; Floyd et al., 2018). Within this body of literature, it has been shown that in students from Thailand and Japan, positive emotional states were correlated with gratitude expressions (both verbal and facial) as well as prosocial behavior. However, only in Japanese male students was there a positive correlation between feelings of indebtedness and increased prosocial motivation (Naito et al., 2005). It seems that a side effect of scholarly focus on gratitude expression may be the current lack of research exploring how feelings of gratitude (as opposed to expressions of gratitude) are associated with other psychological constructs.

Along with the development of the field of positive psychology, there has been significant progress in understanding the biological underpinnings of gratitude, the various psychological benefits it may hold, and methods of cultivating feelings of gratitude in daily life. Recent cross-cultural findings have revealed that gratitude is associated with four different measures of life satisfaction in both Japan and the United States (Robustelli and Whisman, 2018). Although gratitude seems to be correlated with enhanced well-being and may also be linked to a variety of other positive interpersonal and mental health outcomes, these associations have not been systematically compared across cultures. As a result, a cross-cultural nomological network of gratitude in the context of health and well-being is not well established. The current study addresses this limitation by examining the association between gratitude and several domains of health and well-being, including control perception, sympathy, loneliness, life satisfaction, and support/strain from relationships, using data from the United States and Japan. Because the two groups represent contrasting cultural values (i.e., individualism and collectivism), congruence in the patterns among variables would increase confidence that the results are not merely specific to a particular culture. To our knowledge, there has been no empirical investigation into the cross-cultural nomological network of gratitude in relation to various constructs associated with both positive and negative psychological functioning. Illuminating the nature of gratitude in diverse cultural contexts may serve to elucidate similarities and differences in human emotional experience.

## Literature Review

### Conceptualizing Dispositional Gratitude

The word “gratitude” triggers an instinctive understanding for most individuals. Prior work suggests that the concept of gratitude is an intrinsic component of the human experience, entrenched in evolutionary history. Scholars examining gratitude from a neuroscientific perspective have identified brain regions involved in the experience and expression of gratitude. Using functional magnetic resonance imaging (fMRI), it has been shown that the experience of emotional states such as gratitude and pride, which are associated with social values, increased neural activation in the mesolimbic forebrain, a region implicated in reward processing and social bonding (Zahn et al., 2009). Neuroimaging techniques have also demonstrated that a gratitude writing intervention was positively correlated with greater and lasting neural sensitivity to gratitude (Kini et al., 2016). Gratitude has been shown to be associated with neural activation in the medial prefrontal cortex and anterior cingulate cortex, regions recruited during value judgment and moral cognition (Fox et al., 2015).

A variety of frameworks have been developed by researchers in order to conceptualize gratitude and conduct empirical investigations into how it might manifest in daily life. It has been defined as an emotional reaction of appreciation and a universal tendency to have a positive reaction to another’s benevolence (Emmons and Stern, 2013). Emmons and McCullough (2003) define gratitude as “the perception of a positive personal outcome, not necessarily deserved or earned, that is due to the actions of another person.” They further conceptualize it as a two-fold process: (1) “recognizing that one has obtained a positive outcome” and (2) “recognizing that there is an external source for this positive outcome.” Most conceptualizations of gratitude are typically variations on this theme, describing gratitude as a state of appreciation and thankfulness for what is valuable and meaningful to oneself. At the dispositional (trait) level, gratitude refers to a general propensity for being appreciative of positive occurrences (Wood et al., 2010). On the other hand, gratitude as an emotion is temporally specific and involves appreciating the valuable actions of other people, and therefore occurs after receiving aid that is particularly perceived as helpful, altruistic, and costly (McCullough et al., 2001). Scholars have also suggested that when gratitude is defined as an emotion, it should induce prosocial behavior especially when receiving aid from others (McCullough et al., 2001).

Having a grateful disposition has been shown to be associated with a plethora of psychological benefits. Previous research has suggested that trait gratitude is predictive of both life satisfaction and emotional well-being (Wood et al., 2008). Gratitude directed at someone or something is correlated with positive affect and increased emotional well-being (Fredrickson and Joiner, 2002). Furthermore, Breen et al. (2010) found that gratitude and forgiveness are linked to character strengths which in turn are related to positive psychological processes. Individuals with higher trait gratitude are also more likely to exhibit higher levels of self-esteem, optimism, agreeableness, extraversion, conscientiousness, and open-mindedness (Wood et al., 2008; Kong et al., 2015).

Furthermore, prior studies have revealed that a grateful attitude is negatively correlated with harmful psychological tendencies such as avoidance and revenge motivations (Szcześniak and Soares, 2011). Hartanto et al. (2019) found that higher trait gratitude weakens the negative effect of socioeconomic status on health outcomes. Such empirical investigations hold a variety of practical implications in research as well as clinical settings. Certain intervention studies have been highly effective in promoting gratitude, thereby enhancing life satisfaction and subjective well-being (Emmons and McCullough, 2003; Watkins et al., 2003; Lambert et al., 2009; Killen and Macaskill, 2015).

### Cultural Factors Linked to Gratitude

Whereas the majority of empirical studies on gratitude and psychological health have focused on Western individualistic cultures (e.g., the United States), very little research has examined the relationship between gratitude and associated constructs in cultures with collectivistic orientations (Sun et al., 2014; Chen et al., 2015). In a study focusing on physical health effects of trait gratitude among Japanese adults, Boylan et al. (2017) found that gratitude as part of interdependent well-being was not significantly associated with healthy glucoregulation. In a rare cross-cultural study on gratitude, Robustelli and Whisman (2018) examined the role of gratitude on life satisfaction using the MIDUS and MIDJA datasets, and found that gratitude was uniquely and positively associated with satisfaction with relationships and life overall, but not with satisfaction with work or health, after adjusting for demographics and personality characteristics.

Such studies are critical to gaining a deeper understanding of the diversity of emotional experience since cultural upbringing shapes people’s experiences and expressions of gratitude (Floyd et al., 2018). Based on self-report methods, it has been shown that compared to German men, American men experienced grateful feelings less often and sometimes preferred hiding their gratitude, suggesting the presence of cultural variations in internal experiences of gratitude (Sommers and Kosmitzki, 1988). Developmental studies have shown that children from different cultural upbringings begin to express gratitude at different ages and to different extents (Tudge et al., 2016; Payir et al., 2017). Furthermore, compared to individuals in the United States, those in the United Kingdom were more likely to report gratitude as being tied to negative emotions such as indebtedness, embarrassment, guilt, and awkwardness (Morgan et al., 2014). These findings suggest that gratitude may be composed of a common core with culturally ubiquitous elements, as well as socially dependent features that vary depending on the cultural environment (Morgan et al., 2014).

Previous cross-cultural research has also demonstrated that the success of psychological interventions may depend on social and cultural factors. For example, when white Americans participated in an intervention involving writing letters of appreciation to family members or friends, they experienced increased life satisfaction compared to Asian American participants (Boehm et al., 2011). Such findings support the notion that individualistic cultures place more emphasis on self-improvement, thereby reinforcing Western participants’ efforts to experience greater life satisfaction. As a result, this type of intervention may be less efficacious for individuals in collectivistic cultures, which tend to minimize the value of personal agency. For instance, when using a gratitude letter writing intervention, it has been shown that compared to American college students, South Korean college students experienced reduced benefits (Layous et al., 2013). Furthermore, the correlation between effort in the intervention and enhanced well-being was more robust in the American participants. This could have been due to South Korean participants being more likely to feel mixed emotions (e.g., indebtedness in addition to gratitude) while participating in the activity.

The United States and Japan are ideal for comparison in cross-cultural investigations because they are, for the most part, economically alike. Collectivistic cultures like Japan typically promote the maintenance of interpersonal harmony, whereas individualistic cultures such as the United States value independence and personal achievement (Hofstede, 1980; Triandis, 1995; Kitayama et al., 2000, 2009). According to Markus and Kitayama (1991), one’s cultural orientation influences communicative, cognitive, emotional, motivational and behavioral outcomes. The divergent cultural contexts provided by Japan and the United States make the two countries an ideal starting point for testing cross-cultural differences in the relationship between gratitude and its association with other psychological constructs. The fundamental differences in cultural values may underlie variations in levels of gratitude and the correlations with particular domains of psychological health.

To our knowledge, there has been no empirical research investigating the influence of culture on the nomological network of the trait gratitude construct. To address this gap, the current study systematically investigated cross-cultural similarities and differences in correlations between gratitude and various domains of psychological health. A nomological network is invaluable in this regard, as it serves as a theoretical framework representing the fundamental qualities of a construct and the relationships among those basic features. As such, the meaning of a construct lies in the collective body of empirical and theoretical associations with other constructs (Cronbach and Meehl, 1955). Examining cross-cultural consistency (or lack thereof) in the nomological network of gratitude is crucial due to its possible impact on the development of new interventions targeting individuals from diverse ethnic and cultural backgrounds. Furthermore, articulating a nomological network of gratitude has important implications for future empirical investigations, because it allows the subsequent development of novel measures and theoretical frameworks. In addition to the important goal of addressing concerns regarding construct validity, such research will shed light on how the concept of gratitude aligns with the nomological network of other, more established indices of psychological well-being.

The present investigation aims to examine both the convergent and discriminant construct validity of a measure of gratitude. In doing so, gratitude is placed within the broader nomological network of individual level variables that are associated with psychological traits. Whether gratitude predicts theoretically expected outcomes in different cultures is a key issue concerning both its construct validity as well as its research and clinical value.

### Associated Constructs

The present study included all of the common variables that were measured in Japan and the United States, which resulted in the following seven domains.

#### Satisfaction With Life

Previous research has found that gratitude is correlated with general life satisfaction as well as satisfaction across various forms of interpersonal relationships (Froh et al., 2009; Hill and Allemand, 2011; Robustelli and Whisman, 2018). Prior studies have also demonstrated that gratitude is associated with marital satisfaction (Gordon et al., 2012; Algoe and Way, 2014). Gratitude was a direct mediator in the relationship between materialism and decreased life satisfaction among American college students (Tsang et al., 2014).

#### Stress and Social Anxiety

Stress has long been a focal research topic in psychology as well as translational science and medicine due to its association with a plethora of health outcomes and illnesses including depression, cancer, and cardiovascular disease (Cohen et al., 2007). The concept of stress has been empirically examined in three overarching domains: (a) environmental, assessing stressful life occurrences; (b) psychological, focusing on stress appraisal and emotional reactivity; and (c) biological, examining the physiological processes underlying the stress response (Kopp et al., 2010).

Social anxiety is associated with social, educational and work-related functioning impairment (Liebowitz et al., 1985). All facets of life that require interpersonal communication can be hindered by social anxiety disorder, such as maintaining relationships, attending meetings, or joining social groups (Schneier et al., 1994). Social anxiety disorder also has a high comorbidity with other psychiatric disorders, such as depression and alcoholism (Schneier et al., 1992). Petrocchi and Couyoumdjian (2016) found that gratitude served as a protective factor against psychopathology and predicted lower rates of depression and anxiety among Italian participants.

#### Anger Expression

The most widely used strategies of anger regulation as described by Spielberger (1996) are Anger-In, Anger-Out, and Anger-Control. Anger-In (i.e., Anger Suppression) refers to the behavioral tendency to turn feelings of anger inward, or attempting to regulate anger using suppression (Greenglass, 1996). Anger-In has also been defined as the frequency at which an individual experiences feelings of anger without expressing them (Spielberger, 1996). Anger-In is linked to several adverse psychological outcomes such as irritability, depressive symptoms, guilt, and decreased life satisfaction (Gross and John, 2003). In the United States, anger suppression is related to psychological problems that result in decreased life satisfaction. This could be related to the fact that in individualistic cultures, it is often more or less acceptable to express some anger when communicating one’s personal autonomy (Boiger et al., 2013b). Evidence from Japan regarding anger expression seems more complicated. Anger expression as a result of frustration seems to be socially inappropriate in Japan since it interferes with interpersonal harmony, which is highly valued and regarded as a priority in social settings (Boiger et al., 2013a).

#### Sympathy

Scholars have used the term sympathy to refer to two related, but discrete, affective experiences. It has been conceptualized as the experience of feeling the same emotional or affective state as another individual, but also as a feeling of concern for someone in need. However, this same emotional experience has also been labeled as empathy or empathic concern, rather than sympathy (Batson, 2011). Variability in using the two terms has resulted in some inconsistencies in the psychological literature. Previous empirical investigations suggest that the experience of sympathy (empathic concern) arises during perspective-taking, especially of an individual in need, and often leads to altruistic motivation. Furthermore, compared to empathy, sympathy has been shown to be more strongly associated with prosocial behavior (Walter, 2012).

#### Loneliness

Loneliness refers to the subjective perception of detachment from social relationships, and differs from the objective measure of social isolation (Holt-Lunstad et al., 2010). In the United States and Europe, prevalence estimates of loneliness range from 7–39% of adults (Theeke, 2010). Loneliness is correlated with overall functional decline, as well as depression and high blood pressure (Holt-Lunstad et al., 2010). Middle age may be a time in life when individuals are particularly prone to feelings of loneliness, as they face many challenges involving changes in family structure, occupation, and health. As a result, middle-aged adults are exposed to a plethora of stressors which can have negative repercussions on their psychological health and well-being (Antonucci et al., 2001). Previous research has revealed that compared to lonely older adults, lonely middle-aged adults have a higher probability of all-cause mortality (Holt-Lunstad et al., 2010). Studies of middle-aged adults have also found weak social relationships (resulting in loneliness as well as social isolation) to increase vulnerability to stroke and heart disease. However, such studies seldom focus on participants from different countries, and as a result, additional research must be conducted before generalizing to other cultural groups (Antonucci et al., 2001; Holt-Lunstad et al., 2010).

#### Self-Control

Self-control is conceptualized as the ability to resist immediate gains in favor of long-term gratification. It is a cognitive attribute that is discussed within the literature using a variety of terms, such as self-efficacy, mastery, personal control, instrumentalism, self-directedness, locus of control orientation, personal autonomy, and sense of control. Although these terms refer to distinct constructs, they are often used interchangeably within the psychological literature (Skinner, 1996). Previous research has shown that subjective feelings of self-control affect a diverse range of health and relational outcomes, including emotional well-being and increased satisfaction in interpersonal relationships (Tangney et al., 2004). On the other hand, diminished levels of self-control may result in adverse behavioral outcomes including anxiety, conflict in social relationships, drug addiction (as well as other substance use problems), and difficulty maintaining a healthy weight. Nevertheless, self-control seems to be a quality that can be cultivated, as prior studies have demonstrated that positive affect, high cognitive capacity, and willful motivation can increase one’s success at self-control.

#### Relational Support and Strain (Family, Friends, and Spouse)

Social support is commonly understood as one’s perception of the physical and emotional comfort received from one’s relationships (Schuster et al., 1990). In previous research, social support has been measured in various ways, including support received from contacts, perceived availability of support, frequency of network contact, and density of network (Antonucci and Jackson, 1987). Social support has been shown to be essential for maintaining psychological well-being and physical health (Antonucci and Jackson, 1987). Although some studies have attempted to take into account the possible negative influences of interpersonal relationships (i.e., social strain), it is still unclear whether social strain has stronger or weaker repercussions compared to social support. Furthermore, the composition of the network may affect psychological outcomes, as family, friends, and partners may affect social support and strain to different extents. It has been shown that strained interpersonal exchanges and network dissatisfaction are linked to depression and psychological distress (Schuster et al., 1990). Furthermore, prior evidence suggests an association between social strain and physical health measures such as impaired cardiovascular and immune functioning (Kiecolt-Glaser et al., 1993).

## The Current Study

Our study examined whether trait (dispositional) gratitude is associated positively or negatively with a variety of psychological traits in cognitive, affective, and health domains in the United States and Japan. Our objective was to investigate whether a self-reported measure of gratitude is related to a wide range of health-related psychological traits in theoretically expected directions, and whether these trends are consistent across cultures.

We hypothesized that gratitude would be associated with adaptive psychological traits in middle-aged adults in Japan and the United States, since individuals with increased trait gratitude are more likely to report being happy, optimistic, and having increased levels of self-esteem (Kong et al., 2015). Furthermore, a grateful attitude has previously been shown to be correlated with emotional well-being and prosocial motivation (McCullough et al., 2002). On the other hand, prior evidence has suggested that gratitude is negatively correlated with resentment, depression, anxiety, and other measures of maladaptive psychological traits (McCullough et al., 2002, 2004; Lambert et al., 2009). Based on these previous studies, we predicted that a similar pattern of associations between gratitude and psychological health would emerge across cultures.

However, differences could also be expected between Japan and the United States in terms of self-reported levels of gratitude, psychological health constructs, and the robustness of the correlations between variables. Since a grateful disposition may be more aligned with interpersonal harmony as compared to independence, autonomy, and personal success, we expected that Japanese participants would report higher levels of gratitude compared to American participants. Exploratory analyses were also conducted in order to assess the relative strengths of correlations between gratitude and psychological health variables. However, due to the scarcity of literature evaluating these associations across different cultural contexts, we did not make specific predictions about each of the variables.

## Materials and Methods

### Participant Selection

The participants for the current study consisted of adults living in Japan and the United States. The American sample was drawn from the Midlife in the United States (MIDUS Refresher; Weinstein et al., 2016): Biomarker Project, 2012–2016, a national survey of middle-aged adults, which collected data on social, psychological, and physiological measures. The American sample (*N* = 863) comprised of 413 males and 450 females, mean age = 50.84, *SD* = 13.41. The Japanese sample was drawn from the Survey of Midlife in Japan (MIDJA 2, Ryff et al., 2012), which was a parallel dataset of MIDUS and consisted of Japanese-speaking adults from the Tokyo metropolitan area. The Japanese sample (*N* = 657) consisted of 309 males and 348 females, mean age = 59.25, *SD* = 13.55.

### Measures

#### Gratitude

Levels of gratitude were measured using the Gratitude Questionnaire (McCullough et al., 2002), in which the respondents were asked to rate how they evaluate their life overall, and how much they agree (or disagree) with the following 2 items: (1) “I have so much in life to be thankful for” and (2) “I am grateful to a wide variety of people.” Their responses were coded on a 7-point scale ranging from 1 (strongly disagree) to 7 (strongly agree), with the higher scores representing higher levels of gratitude. The correlations between the two items were: *r* = 0.58 in the United States and *r* = 0.81 in Japan.

#### Life Satisfaction

Pavot and Diener’s (1993) scale was used to measure satisfaction with life overall. It had five items including: (a) “In most ways my life is close to my ideal.” (b) “The conditions of my life are excellent.” (c) “So far I have gotten the important things I want in life.” The items were assessed on a 7-point scale, ranging from 1 (Strongly disagree) to 7 (Strongly agree). Cronbach’s alphas were 0.89 (United States) and 0.90 (Japan).

#### Social Anxiety

The Liebowitz Social Anxiety Scale examines levels of social anxiety along with a variety of psychometric properties (Fresco et al., 2011). It had nine subscale items including: (a) speaking with figures of authority; (b) attending a party; (c) working under supervision; (d) speaking with unfamiliar people; (e) being the center of attention. Items were assessed on a 4-point scale ranging from 1 (none) to 4 (severe). Cronbach’s alphas were 0.85 (United States) and 0.90 (Japan).

#### Perceived Stress

The Perceived Stress Scale by Cohen et al. (1983) was used to measure levels of stress. It consisted of 10 items, including: In the last month, how often have you … (a) been upset due to a unexpected occurrence; (b) felt agitated; (g) been able to control annoyances in life; (i) been angered due to things that aren’t within your control; and (j) felt that difficulties were accumulating to an overwhelming level. Responses were assessed using a 7-point scale ranging from 1 (strongly disagree) to 7 (strongly agree). Cronbach’s alphas were 0.86 (United States) and 0.79 (Japan).

#### Anger Expression

Anger expression was assessed using the Anger-In, Anger-Out, and Anger-Control measures from the State-Trait Anger Expression Inventory (STAXI; Spielberger, 1996). The three subscales refer to the extent to which “one can keep angry feelings inside or can suppress anger or furious feelings,” “one can express feelings of anger, furious feelings, or lose control,” and “one can control anger or furious feelings using physical or verbal expression and communication,” respectively. Items were measured using a 4-point scale, ranging from 1 (almost never) to 4 (almost always). Sample items included: In general when I feel angry, I withdraw from people (Anger-In); I express my anger (Anger-Out); and I control my temper (Anger-Control). Cronbach’s alphas of Anger-In were 0.82 (United States) and 0.75 (Japan), Anger-Out were 0.79 (United States) and 0.83 (Japan), and Anger-Control were 0.67 (United States), and 0.70 (Japan).

#### Sympathy

Levels of sympathy were measured using the Sympathy Scale (Uchida and Kitayama, 2001) in which respondents were asked to rate how their views of themselves are linked to their relations with others, based on the following 4 items: (a) “Even when things are going well for me, I can’t be happy if I have a friend who is in trouble”; (b) “I am moved when I hear of another person’s hardship”; (c) “I think that nothing is more important than to be sympathetic to others”; and (d) “My sympathy has its limits” (reversed item). Their responses were again coded on a 7-point scale ranging from 1 (strongly agree) to 7 (strongly disagree). Alphas were 0.50 (United States) and 0.54 (Japan).

#### Loneliness

Levels of loneliness were assessed by the shortened 7-item version of the UCLA Loneliness Scale developed by Russell (1996). The scale assessed how often a person felt disconnected from others and perceived a lack of support and social companionship from their interpersonal relationships. Sample items included: (a) “There is no one I can turn to”; (b) “No one really knows me well”; (c) “I feel isolated from others.” Alphas were 0.87 (United States) and 0.79 (Japan).

#### Self-Control

Self-control was measured using the Cognition Control, Emotion Control, and Burden Consciousness subscales of the Self-control Scale (Markus and Kitayama, 1991; Gross and John, 2003). The six cognition control items, six emotion control items, and seven burden consciousness items were coded using a 7-point scale, ranging from 1 (strongly agree) to 7 (strongly disagree). Sample items are as follows: (a) “I can make myself do things I don’t want to do” (Cognition Control); (b) “I control my emotions by changing the way I think about the situation I’m in” (Emotion Control); (c) “It is important to me that I not bother others” (Burden Consciousness). Alphas ranged from 0.60 to 0.69 (United States), and from 0.53 to 0.79 (Japan).

#### Friend Support

This scale was based on a study by Schuster et al. (1990). Friend Support was assessed by four items, including “How much do you really care about your friends?” It was coded on a scale of 1 (Not at all) to 4 (A lot). Friend Strain was measured by four items, including “How often do you make too many demands on your friends?” Responses were coded on a scale of 1 (Never) to 4 (Often). A Friend Affectual Solidarity score was obtained based on an eight-item scale combining the four “Friend Support” items and the four “Friend Strain” items. Alphas ranged from 0.64 to 0.72 (United States), and from 0.69 to 0.85 (Japan).

#### Family Support

This scale was based on a study by Schuster et al. (1990) and Walen and Lachman (2000). Family Support was assessed by two items, including “How much can your family (not including your spouse or partner) rely on you for help if they have a serious problem?” It was coded on a scale of 1 (Not at all) to 4 (A lot). Family Strain was measured by four items, including “How often do you make too many demands on members of your family?” Responses were coded on a scale of 1 (Never) to 4 (Often). An Affectual Solidarity Given to Family score was obtained based on an six-item scale combining the two “Family Support” items and the four “Family Strain” items. Alphas ranged from 0.57 to 0.70 (United States), and from 0.58 to 0.85 (Japan).

#### Spouse/Partner Support

This scale was based on a study by Schuster et al. (1990), Grzywacz and Marks (2000), and Walen and Lachman (2000). Spouse/Partner Support was assessed by six items, including “How much do you really care about your spouse/partner?” It was coded on a scale of 1 (Not at all) to 4 (A lot). Spouse/Partner Strain was measured by six items, including “How often do you make too many demands on your spouse/partner?” Responses were coded on a scale of 1 (Never) to 4 (Often). A Spouse/Partner Affectual Solidarity score was obtained based on a twelve-item scale combining the six “Spouse/Partner Support” items and the six “Spouse/Partner Strain” items. Alphas ranged from 0.79 to 0.82 (United States), and from 0.84 to 0.88 (Japan).

## Results

### Preliminary Analyses

Before testing the associations between gratitude and domains of psychological traits, we examined the differences in variable means between the United States and Japan. Means, standard deviations, *t*-test results, and effect sizes [Cohen’s *d*] are listed in Table 1. Independent samples *t*-tests were conducted for the two countries to investigate mean differences in gratitude levels and associated constructs.

**TABLE 1:** Descriptive statistics for all variables.

			Japan							USA					Mean difference		
	
	*M*	*SD*	*Min*	*Kurt.*	*Skew.*	*N*	*M*	*SD*	*Min*	*Max*	*Kurt.*	*Skew.*	*N*	*t*	*df*	*P*	*d*
Gratitude	5.55	1.04	1.00	1.16	–0.78	653	6.18	0.90	1.00	7.00	3.13	–1.46	860	12.60	1511	P<.001	–0.66
Satisfaction with Life	4.08	1.18	1.00	0.24	–0.37	653	4.68	1.34	1.00	7.00	–0.40	–0.49	860	9.08	1511	p-<.001	–0.47
Stress & Anxiety																	
Percieved Stress	26.10	5.70	10.00	0.71	0.20	643	22.49	6.36	10.00	44.00	–0.09	0.47	861	11.38	1502	p<.001	0.59
Social Anxiety	1.79	0.54	1.00	0.74	0.69	643	1.84	0.55	1.00	4.00	0.01	0.61	862	1.63	1503	*n.s.*	–0.09
Anger Expression																	
Anger/In	14.01	3.62	8.00	0.86	0.72	647	15.39	4.48	8.00	30.00	–0.06	0.58	862	0.42	1507	*p*<.001	–0.34
Anger/Out	12.07	3.50	8.00	3.07	1.36	644	13.40	3.55	8.00	31.00	1.97	1.07	862	7.24	1504	p<.001	–0.38
Anger/Control	7.98	2.47	4.00	–0.58	0.20	639	9.94	2.21	4.00	15.00	–0.65	–0.30	862	16.15	1499	p<.001	–0.84
Adjustment	1.81	0.74	1.00	0.60	0.92	645	2.04	0.67	1.00	4.00	0.24	0.53	862	6.30	1505	p<.001	–0.32
Self-Construal																	
Interdependence	4.71	0.63	1.42	1.48	–0.29	642	5.12	0.67	1.00	7.00	2..30	–0.63	860	12.03	1500	p<.001	–0.64
Independence	4.73	0.65	1.40	1.40	–0.16	642	5.29	0.81	1.00	7.00	0.68	–0.46	860	14.43	1500	p<.001	–0.75
Sympathy																	
Sympathy	5.01	0.62	2.40	0.69	–0.06	646	4.65	0.62	2.30	6.60	0.37	–0.25	859	11.16	1503	p<.001	0.59
Loneliness																	
UCLA Loneliness	13.61	3.99	7.00	–0.45	0.06	639	12.68	4.49	7.00	28.00	–0.20	0.70	861	4.10	1498	p<.001	0.22
Self-control																	
Self-control	4.74	0.53	2.83	0.42	0.10	648	4.96	0.54	3.11	6.58	0.11	–0.04	860	7.89	1506	p<.001	–0.42
Cognition Control	4.83	0.74	2.20	0.60	–0.02	651	5.36	0.75	1.00	7.00	1.53	–0.59	860	13.68	1509	p<.001	–0.71
Emotion Control	4.68	0.75	1 17	1.22	–0.23	652	4.69	0.83	1.33	7.00	(1.19	–0.14	860	0.24	1510	n.s.	–0.01
Burden Consciousness	4.70	0.61	2.57	0.42	0.08	650	4.85	0.77	1 4.3	7.00	0.45	–0.18	860	4.09	1508	p<.001	–0.21
Friends																	
Support Given to Friends	2.48	0.55	1.00	0.47	0.03	650	3.62	0.40	1.00	4.00	5.31	–1.78	860	46.62	1508	p<001	–2.43
Strain Given to Friends	1.66	0.47	1.00	–0.42	0.12	649	1.58	0.45	1.00	4.00	2.86	1.14	857	3.35	1504	p<.01	0.19
Affectual Solidarity Given to Friends	2.91	0.33	1.75	0.38	0.35	648	3.52	0.32	1.75	4.00	2.41	–1.12	857	36.13	1503	p<.001	–1.91
Family																	
Support Given to Family	132	0.71	1.00	–0.07	0.30	639	3.72	0.52	1.00	4.00	8.27	–2.60	860	44.07	1497	p<.001	–2.30
Strain Given to Family	1.83	0.54	1.00	–0.09	0.13	639	1.75	0.49	1.00	4.00	1.40	0.84	857	2.99	1494	p <.01	0.15
Affectual Solidarity Given to Family	2.88	0.40	1.50	0.67	0.09	640	3.40	0.38	1.50	4.00	1.49	–0.95	857	25.61	1495	p<.001	–1.33
Spouse																	
Support Given to Spouse	2.88	0.58	1.00	–0.17	–0.09	466	3.81	0.34	1.50	4.00	14.98	–3.31	560	31.92	1024	p<.001	–1.99
Strain Given to Spouse	2.13	0.49	1.00	0.27	–0.19	466	2.05	0.5.5	1.00	4.00	0.44	0.51	559	2.49	1023	p<.05	0.17
Affectual Solidarity Given to Spouse	2.87	0.42	1.50	0.31	0.22	466	3.38	0.36	1.67	4.00	2.28	–1.13	559	20.93	1023	p<.001	–1.31

**M, mean score; SD, standard deviation; Min, minimum score; Max, maximum score; Kwrt., kurlosis; Skew., skewness; N, number of participants; t, t-value; df, degree of freedom; p, probability; d, effect size. All variables were calculated using listwise deletion. The proportion of missing values is quite small except for the variables involving “Spouse” (Support Given by Spouse, Strain Given by Spouse, and Affectual Solidarity Given by Spouse), which can be explained by some participants not having a spouse.**

A series of *t*-tests revealed several significant differences in levels of gratitude and life satisfaction between the United States and Japan. Compared to the Japanese participants, American participants reported higher mean levels of gratitude. Given the differences in cultural values and societal expectations between the United States and Japan, one would expect that Japanese participants would have higher mean levels of gratitude, since cultures with collectivistic orientations are more likely to prioritize social harmony over personal autonomy. However, mean levels of gratitude may have been elevated in the United States due to social desirability effects. This may lead to a tendency to report prosocial and altruistic dispositions that align with cultural values (Wood et al., 2010).

In terms of effect sizes, Cohen’s *d* is considered to be small (0.2), medium (0.5), and large (0.8) when interpreting the magnitude of the differences. In this study, out of the 22 variables, 11 variables revealed a Cohen’s *d* effect size of over 0.50 (Gratitude, Perceived Stress, Anger Control, Sympathy, Cognition Control, Support Given to Friends, Affectual Solidarity Given to Friends, Support Given to Family, Affectual Solidarity Given to Family, Support Given to Spouse, and Affectual Solidarity Given to Spouse). As compared to the Japanese participants, Americans reported higher levels of Gratitude, Satisfaction with Life, Anger Control, Cognition Control, Support Given to Relational Network, as well as Affectual Solidarity Given to Relational Network. On the other hand, Japanese participants reported higher levels of Perceived Stress and Sympathy.

### Measurement Invariance Test of Gratitude Factors

In order to check the equivalence of the Gratitude factor between the United States and Japan, we conducted a measurement invariance test. Because the gratitude scale has only two items, it was not possible to compare the factor structure between United States and Japan. Therefore, we conducted multi-group structural equation modeling using two-factor structure of the two-item Gratitude scale and the Gratitude factor of the Minimalist Well-being scale. We set two Gratitude factors (the two-item scale and the Minimalist scale with a covariance between them) and examined the measurement invariance of those scales between the United States and Japan. We tested for measurement invariance in the following order. Model 1 tests configural invariance which represents a common factor structure between the countries. Model 2 tests metric invariance that has same factor loadings between the groups. Model 3 tests scalar invariance that has same intercepts of indicators between the groups. Fit indices of the three models were as follows: For Model 1, χ^2^ = 140.194, df = 26, *p* < 0.001, CFI = 0.971, RMSEA = 0.054, AIC = 228.194; For Model 2, χ^2^ = 240.356, df = 33, *p* < 0.001, CFI = 0.947, RMSEA = 0.064, AIC = 314.356; For Model 3, χ^2^ = 714.474, df = 40, *p* < 0.001, CFI = 0.827, RMSEA = 0.105, AIC = 774.474. We found that Model 1 provided the best fit, and that the difference of CFI between Model 1 and Model 2 was more than 0.01.

Since these results only support configural invariance, we conducted partial metric invariance tests. We found that the model showed the best fit when only two of the five paths were restricted to be the same in the Gratitude factor of the Minimalist Well-being scale: χ^2^ = 183.285, df = 30, *p* < 0.001, CFI = 0.961, RMSEA = 0.058, AIC = 263.285. The difference in CFI between Model 1 and the final model was 0.01. Therefore, equivalence can be assumed since the difference in CFI is less than or equal to 0.01 (Cheung and Rensvold, 2002). In the final model, the path coefficients of the two-item Gratitude scale were set as the same, supporting the metric invariance of the scale. In addition to the final model, we tested an additional model in which a coefficient of covariance was the same between the United States and Japan: χ^2^ = 183.975, df = 31, *p* < 0.001, CFI = 0.961, RMSEA = 0.057, AIC = 261.975. The fit indices of the additional model were nearly identical to the final model, suggesting that the covariance between the Gratitude factors is equivalent between the United States and Japan (*r* = 0.634, *p* < 0.001).

### Main Analyses

We tested for differences and similarities between the United States and Japan in regard to the robustness of the correlations between gratitude and the psychological variables. First, Pearson correlation coefficients were computed for the variables (see Table 2). To rule out potential response bias among participants in the United States and Japan, we created the variables based on standardized item scores and calculated correlation coefficients between gratitude and other variables for the United States and Japan, and *z*-values for the difference of correlation coefficients between the United States and Japan. Overall, we found quite similar patterns of relationships among the variables based on unstandardized scores and standardized scores. Therefore, bivariate correlations (based on unstandardized scores) between gratitude and other psychological traits were then examined. The patterns of relationships were mostly consistent across the United States and Japan; that is, the *z*-scores (for comparing the magnitude of differences in correlation coefficients) were significant for only 7 variables out of 21. In Table 2, we reported partial correlation coefficients after controlling for gender and age. The results are nearly identical to zero-order correlations.

**TABLE 2:** Correlation coefficients and partial coefficients (controlling for age and gender).

	Gratitude			Gratitude	
	Japan r	USA r	Difference z	Japan r *partial*	USA r *partial*
Gratitude					
Satisfaction with Life	0.46***	0.46***	0.00	0.45***	0.46***
Stress & Anxiety					
Percieved Stress	−0.22***	−0.30***	1.64	−0.24***	−0.31***
Social Anxiety	−0.11**	−0.17***	1.17	−0.15***	−0.17***
Anger Expression					
Anger/In	−0.14***	−0.14***	0.00	−0.15***	−0.12***
Anger/Out	–0.05	−0.13***	1.55	–0.05	−0.12***
Anger/Control	0.12**	0.14***	–0.39	0.15***	0.14***
Adjustment	–0.03	–0.01	–0.38	–0.01	0.01
Self-Construal					
Interdependence	0.38***	0.28***	2.15*	0.40***	0.28***
Independence	0.27***	0.16***	2.21*	0.30***	0.16***
Sympathy					
Sympathy	0.42***	0.31***	2.44*	0.41***	0.30***
Loneliness					
UCLA Loneliness	−0.37***	−0.45***	1.84	−0.38***	−0.44***
Self-control					
Self-control	0.34***	0.15***	3.84***	0.33***	0.15***
Cognition Control	0.36***	0.32***	0.87	0.36***	0.31***
Emotion Control	0.25***	0.02	4.52***	0.26***	0.03
Burden Consciousness	0.16***	0.00	3.10**	0.13**	–0.01
Friends					
Support Given to Friends	0.33***	0.24***	1.88	0.33***	0.22***
Strain Given to Friends	0.02	−0.08*	1.92	0.03	−0.07**
Affectual Solidarity Given to Friends	0.27***	0.21***	1.22	0.26***	0.20***
Family					
Support Given to Family	0.19***	0.16***	0.59	0.18***	0.15***
Strain Given to Family	0.03	−0.13***	3.07**	0.01	−0.13***
Affectual Solidarity Given to Family	0.10*	0.19***	–1.76	0.10*	0.18***
Spouse					
Support Given to Spouse	0.39***	0.22***	2.99**	0.40***	0.22***
Strain Given to Spouse	0.00	−0.19***	3.06**	0.00	−0.19***
Affectual Solidarity Given to Spouse	0.27***	0.25***	0.34	0.29***	0.25***

*p < 0.0.5, **p < 0.01, ***p < 0.001; Number of participants is the same as Table 1; r = correlation coefficients; r_*partial*_ = partial correlation coefficients controlled with gender and age; z = standardized difference of correlation coefficients.

Of the numerous psychological traits examined, Satisfaction with Life was identified as the strongest positive correlate of trait gratitude in both the United States (0.46) and Japan (0.46). Consistent with prior research, evidence of convergent validity was observed, with moderate-sized negative correlations between Gratitude and Perceived Stress in both countries (*r* = −0.22, *p* < 0.001 for Japan; *r* = −0.30, *p* < 0.001 for the United States) and Social Anxiety (*r* = −0.11, *p* < 0.001 for Japan; *r* = −0.17, *p* < 0.001 for the United States). Among the three styles of Anger expression, positive associations were observed between the Gratitude measure and Anger Control (*r* = 0.12, *p* < 0.001 for Japan; *r* = 0.14, *p* < 0.001 for the United States). On the other hand, higher levels of Gratitude were negatively associated with Anger-In in both countries (*r* = −0.14, *p* < 0.001 for both countries). Anger-Out was negatively associated with the sense of Gratitude only in the United States (*r* = −0.05, NS for Japan; *r* = −0.13, *p* < 0.001 for the United States).

The Gratitude measure was found to be positively associated with Sympathy in both countries (*r* = 0.42, *p* < 0.001 for Japan; *r* = 0.31, *p* < 0.001 for the United States), while it was negatively associated with Loneliness in both countries (*r* = −0.37, *p* < 0.001 for Japan; *r* = −0.45, *p* < 0.001 for the United States). Regarding the Self-Control scale, Cognition Control was positively associated with Gratitude in both countries (*r* = 0.36, *p* < 0.001 for Japan; *r* = 0.32, *p* < 0.001 for the United States). On the other hand, Emotion Control (*r* = 0.25, *p* < 0.001) and Burden Consciousness (*r* = 0.16, *p* < 0.001) were positively associated with Gratitude only in Japan.

Support Given to Relational Network was positively associated with Gratitude in both countries: for Friends, (*r* = 0.33, *p* < 0.001 for Japan; *r* = 0.24, *p* < 0.001 for the United States; for Family, *r* = 0.19, *p* < 0.001 for Japan; *r* = 0.16, *p* < 0.001 for the United States; for Spouse, *r* = 0.36, *p* < 0.001 for Japan; *r* = 0.32, *p* < 0.001 for the United States). On the other hand, Strain Given to Relational Network was negatively associated with Gratitude only in the United States (for Friends, *r* = −0.08, *p* < 0.001; for Family, *r* = −0.13, *p* < 0.001; for Spouse, *r* = −0.19, *p* < 0.001). As expected, the Gratitude measure showed moderately sized positive correlations with Affectual Solidarity both in the United States (for Friends, *r* = 0.21, *p* < 0.001; for Family, *r* = 0.19, *p* < 0.001; for Spouse, *r* = 0.25, *p* < 0.001) and in Japan (for Friends, *r* = 0.27, *p* < 0.001; for Family, *r* = 0.10, *p* < 0.001; for Spouse, *r* = 0.27, *p* < 0.001).

## Discussion

The present study aimed to clarify the convergent and divergent validity of the gratitude construct among middle-aged adults living in the United States and Japan. Consistent with our hypotheses, the results revealed significant bivariate correlations between trait gratitude and measures of adaptive psychological traits in both countries. Specifically, self-reported gratitude was most strongly associated with Life Satisfaction, followed by Sympathy, Cognition Control, and Support Given to Relational Network (see Table 2). Consistent with our hypotheses, we also found that gratitude was negatively correlated with measures of maladaptive psychological traits such as Loneliness, Perceived Stress, Social Anxiety, and Anger-In in both the United States and Japan. Strain Given to Relational Network was also negatively associated with trait gratitude in the United States.

These results build upon prior studies by demonstrating that higher levels of gratitude are linked to adaptive psychological traits and enhanced well-being, whereas lower levels of gratitude are related to negative psychological processes and poor emotional health. The observed pattern of results implies that the powerful psychological benefits of a grateful disposition may be consistent across cultures. Prior studies using participants from the United States have also revealed a strong correlation between gratitude and life satisfaction or psychological health (Emmons and McCullough, 2003; Hill and Allemand, 2011). Furthermore, other studies suggest that a grateful disposition increases the likelihood of using active coping styles and social support, resulting in enhanced psychological health in the United States (Lin and Yeh, 2014) and China (Kong et al., 2015). Consistent with results from these past studies, the current study suggests that being grateful is positively associated with life satisfaction, as well as increased sympathy and sense of control.

Results from the current study also showed that Support and Affectual Solidarity Given to Relational Network, which is a combination of Friends, Family and Spouse, was positively associated with gratitude in both the United States and Japan, while Strain Given to Relational Network was negatively associated with gratitude among the American participants only. The fact that Strain Given to Relational Network was not negatively associated with gratitude in Japan may have been due to the possibility that Japanese participants assessed “demands” made to their relational network as not so much a strain, but rather as an expected part of the relationship. Those aspects of interpersonal “strain” may be culturally condoned in Japan, and in fact, be involved in the successful navigation of the social environment. In other words, given the lack of association between Gratitude and Strain Given to Friends/Family/Spouse among Japanese participants, we can cautiously speculate that the interpersonal rules governing one’s obligatory relational network (via relational demands) in Japan may neither dampen nor increase one’s overall sense of gratitude, but may be perceived as cultural/social obligations and a component of individual responsibility in a closely knit society.

In addition, Anger-Out was negatively associated with Gratitude only in the United States (even though there was a weak negative trend of −0.05 in Japan). In the interdependent cultural context of Japan, outward anger expression (Anger-Out) may be especially undesirable due to its deleterious effects on social harmony. Given a very weak association between Anger-Out and Gratitude in Japan, we cautiously speculate that the tendency to express anger (“Anger-Out”) in Japan may be governed primarily by cultural/social sanctions.

Overall, when the nomological network of gratitude was analyzed separately in the United States and Japan, the results were nearly identical. This suggests that across cultures, being grateful generally seems to have similar relationships with a wide variety of psychological variables. Overall, the scale reliabilities of the psychological measures used were strong, and the relationships between gratitude and other variables are consistent with the existing literature on gratitude. This evidence permits cross-cultural validation of the nomological network of gratitude and reduces the likelihood of chance or sample-specific findings. These findings should help establish gratitude as a construct that has consistent relationships with other well-established psychological constructs in both individualistic and collectivistic societies.

The present study is not without limitations. First, the gratitude measure used was a shortened version of the longer scale and only comprised of two items, possibly providing a less reliable and/or valid measure of the gratitude construct compared to more comprehensive gratitude scales. MIDUS studies used an abbreviated, two-item measure of trait gratitude due to their convenience and ease of use. Even though partial metric invariance tests suggest that the covariance between the Gratitude factors is equivalent between the United States and Japan, previous research evaluating the consequences of using short measures of the Big Five personality traits has revealed that the use of very short measures of personality may substantially increase the Type 1 and Type 2 error rates (Credé et al., 2012). Therefore, it may be beneficial to utilize longer measures which can substantially increase the validity of research findings, thereby avoiding potential errors in estimating individual levels of trait gratitude.

Second, this study focused on bivariate associations between trait gratitude and related psychological variables. Thus, it did not address theoretical bases of cultural similarities and differences in associations among these variables including trait gratitude. Using the MIDUS and MIDJA dataset, Kitayama et al. (2010) found that the negative association between relational strain and well-being was stronger among Japanese than Americans. Based on such results, future research should investigate whether trait gratitude may mediate (or moderate) the relationship between relational strain and well-being, which may differ across cultures. Furthermore, Japanese with higher socioeconomic status (SES) expressed more anger, whereas Americans with lower SES expressed more anger (Park et al., 2013). Future cross-cultural research should examine whether trait gratitude may serve as mediator (or moderator) in the relationship between anger expression and relational strain.

Lastly, although our results revealed a similar pattern of associations between countries, further studies are needed to conceptualize and classify different forms of gratitude and related affective experiences in diverse cultural contexts. Future research should continue to shed light on the cultural similarities and differences in the longitudinal and cross-sectional links between gratitude and other well-known psychological constructs. Such lines of investigation could inform research on gratitude interventions and ultimately lead to promising clinical applications.

Our study helps to clarify the relationship between gratitude and other well-known psychological constructs in both Japan and the United States. Our main finding that gratitude is positively associated with adaptive psychological traits across cultures warrants continued investigations of how gratitude interventions can be used effectively across diverse cultural contexts, ultimately enhancing well-being in various domains of life. However, particularly when conducting interventions in collectivistic cultures, it is crucial to acknowledge that gratitude may be experienced along with other feelings, resulting in mixed emotion states. However, conducting interventions in collectivistic cultures, it is crucial to acknowledge that gratitude may be experienced along with other feelings, resulting in mixed emotions (Robustelli and Whisman, 2018). Previous research has suggested that individuals from collectivistic cultural backgrounds may experience a combination of indebtedness and gratitude when others are kind or generous to them (Kim et al., 2006). There is prior evidence suggesting that Asian Americans tend to be more wary of negatively affecting social networks (e.g., others viewing them as a burden) and as a result, may refrain from seeking out social support after experiencing stressful life events (Kim et al., 2006). Future research should investigate whether expressing gratitude may invoke feelings of indebtedness and guilt in the expresser, as a result of owing a favor to another and having to reciprocate an act of kindness or generosity.

In summary, our findings suggest that gratitude is linked to psychological health for individuals in different cultural environments. The importance of further untangling this construct is evident, as it has strong implications for health-related interventions and clinical research that take varied cultural contexts into account. Deepening our knowledge of culture-general versus culture-specific relationships of psychological constructs surrounding gratitude would advance our understanding of human emotional experience. Ultimately this may spur the development of interventions seeking to enhance the psychological well-being of individuals from a wider array of ethnic and cultural backgrounds.

## Data Availability Statement

All relevant data from the Midlife in the United States (MIDUS) Study and the Midlife in Japan (MIDJA) Study can be downloaded for free at http://midus.wisc.edu/data/index.php.

## Ethics Statement

MIDUS and MIDJA data collection was reviewed and approved by the Education and Social/Behavioral Sciences and the Health Sciences IRBs at the University of Wisconsin-Madison.

## Author Contributions

TS and AO contributed to the conception and design of the study. AO performed the statistical analysis. TS, AO, AY, and SA wrote sections of the manuscript. All authors contributed to manuscript revision and read and approved the submitted version.

## Conflict of Interest

The authors declare that the research was conducted in the absence of any commercial or financial relationships that could be construed as a potential conflict of interest.
